# Two years neglected knee dislocation: An unusual case report

**DOI:** 10.1016/j.ijscr.2021.106745

**Published:** 2021-12-30

**Authors:** Hatim Garnaoui, Mohamed ali Trafeh, Charafeddine El Kassimi, Abderrahim Rafaoui, Mohamed Rahmi, Abdelhak Garch

**Affiliations:** Orthopedic Department P32, University Hospital Ibn Rochd, Faculty of Medicine & Pharmacy of Casablanca, Morocco

**Keywords:** Neglected dislocation, Knee, Open reduction, Results

## Abstract

**Introduction:**

Traumatic dislocation of the knee is a rare and severe injury, often caused by a severe trauma. Neglected knee dislocations are much more uncommon and treatment options are not clearly described.

**Presentation of case:**

We reported the case of a 26-year-old man with two years neglected knee dislocation which was managed with open reduction and stabilization with external fixation and intra-articular Steinman pins for six weeks.

**Discussion and conclusion:**

Knee dislocations are rare injuries that can be underestimated during the emergency assessment, particularly in the absence of neurovascular injuries, which usually lead to surgical treatment, or the presence of severe trauma that disguises dislocation as the case for our patient. Only a high clinical suspicion can avoid neglecting the more severe joint injury.

## Highlights

• Traumatic dislocation of the knee is a rare and severe injury, often caused by a severe trauma.
• Knee dislocation requires an emergency treatment to achieve immediate and stable reduction and to treat associated injuries.
• Neglected knee dislocations are much more uncommon and treatment options are not clearly described.
• Only a high clinical suspicion can avoid to neglect the more severe joint injury.
• The treatment options include open reduction and fixation, arthrodesis, and total knee arthroplasty.

## 1 Introduction

Traumatic dislocation of the knee is a rare and severe injury, often caused by a severe trauma. It accounts for 0.02% of all extremity injuries [1]. This number is most likely underestimated due to spontaneous reductions and missed diagnosis [2].

Knee dislocation is an orthopedic emergency, it requires an emergency treatment to achieve immediate and stable reduction and to treat associated injuries, especially neurovascular ones. Neglected knee dislocations are much more uncommon [3]. Because of rarity of the case there is a paucity of reports in the literature, treatment options are not clearly described [4]. We report a case of two years neglected dislocation of the knee after open leg trauma, treated with open reduction.

This case report has been reported in line with the SCARE 2020 criteria [5].

## 2 Case report

A 26-year-old man, presented to our department with complaints of pain and deformity of his right knee and inability to weight-bear on the right leg, resulting from a road accident two years ago causing him an open leg injury, he was treated by an external fixator at another center. The dislocation of the knee had passed unnoticed.

**Fig. 1:**
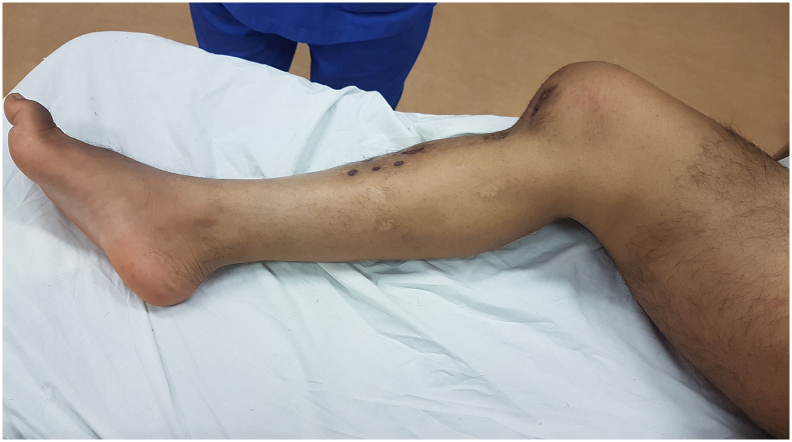
Clinical picture showing the deformity of the knee.

At the clinical exam, he presented fixed posterior knee dislocation, distal end of femur was protruding anteriorly with wasting of thigh muscle. The dislocation was irreducible with no antero-posterior or varus-valgus laxity of the joint. Shortening of the limb was 3 cm and passive range of motion of knee joint was 0 to 30 degrees. The distal neurovascular status however was normal (Fig. 1).

**Fig. 2:**
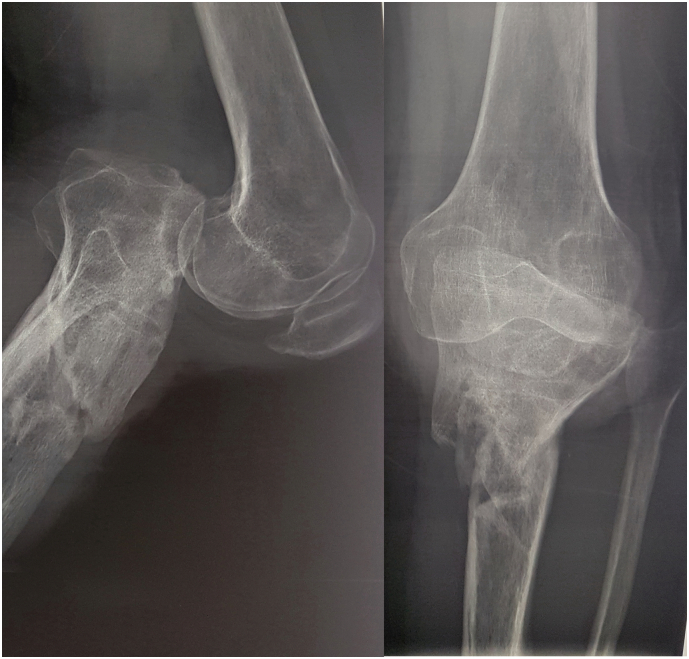
Preoperative knee radiographs showing a posterior knee dislocation with a bone callus on the proximal third of the tibial shaft.

X-rays revealed a posterior knee dislocation with a bone callus on the proximal third of the tibial shaft. A diffuse demineralization was noted (Fig. 2).

Doppler ultrasound of the lower limbs documented no vascular injuries, no intimal narrowing of popliteal, posterior tibial and dorsalis pedis arteries. No deep venous thrombosis was noted.

**Fig. 3:**
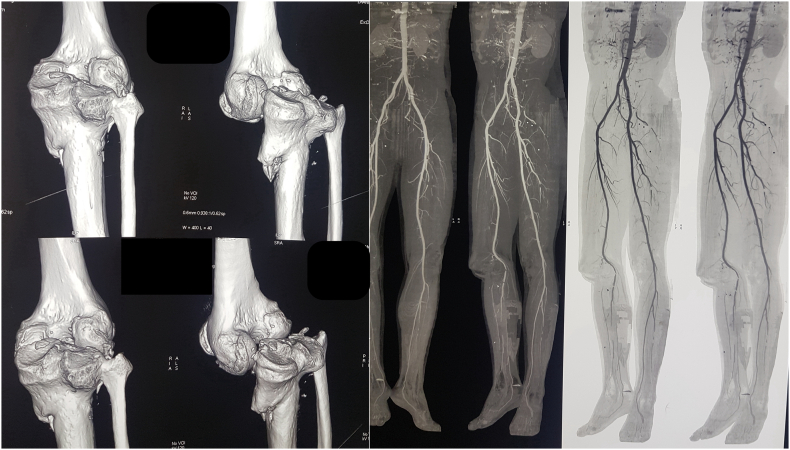
Computed tomography angiography showing normal arterial blood flow.

Computed tomography angiography showed multiple osteochondral lesions and the formation of a pseudo joint. The angiography sequence confirmed normal arterial blood flow thanks to the adaptation of the neurovascular structures to the posterior dislocation (Fig. 3).

**Fig. 4:**
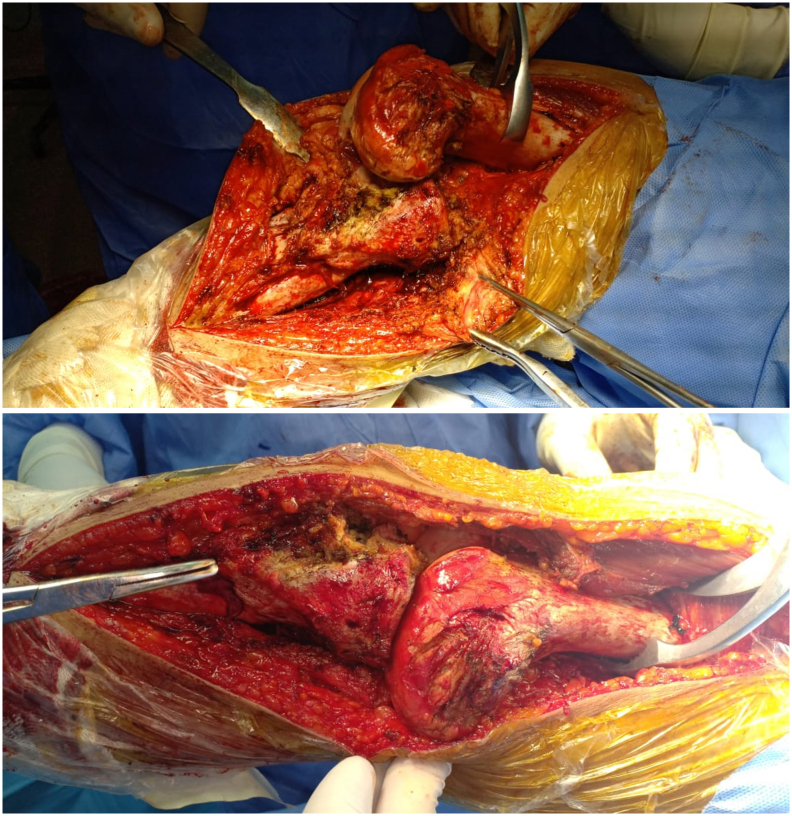
Intraoperative images showing reduction of joint.

**Fig. 5:**
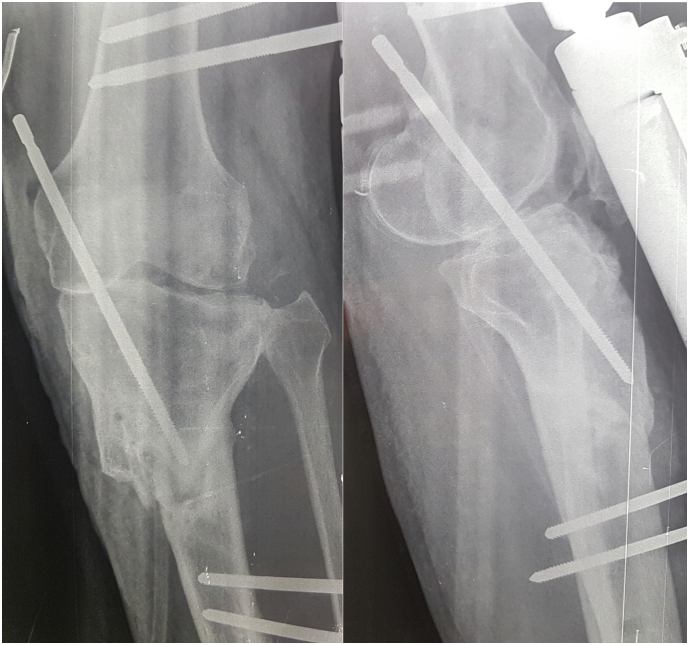
Knee radiographs showing reduction of joint and fixation with orthofix external fixator and an intra-articular Steinman pin.

The patient underwent right knee arthrotomy through a medial para-patellar approach, fibrous adhesions and the extensor mechanism were released to have full exposure of the knee joint. Multiple cartilage lesions were observed and fibrous tissue occupied the inter condylar notch (Fig. 4). After reduction, the joint was stabilized with orthofix external fixator and an intra-articular Steinman pin passed through the medial femoral condyle (Fig. 5). The external fixator and Steinman pin were removed after 6 weeks and knee mobilization was started under supervision with a motion control brace.

At 6 months follow up, the knee range of motion was 0° to 30°, the patient was walking full weight bearing however he experienced mild pain on walking. The pain intensity score was 3 out of 10 on the visual analogue scale.

Regular clinical and radiologic follow ups are planned, and an eventual total knee arthroplasty will be considered depending on the clinical evolution.

## 3 Discussion

Knee dislocations are rare injuries that can be underestimated during the emergency assessment. Dislocation, or subluxation of the knee, can be misdiagnosed at the time of the trauma, particularly in the absence of neurovascular injuries, which usually lead to surgical treatment [6], [7], or the presence of severe trauma that disguises dislocation as the case for our patient.

Most acute knee dislocations are either reduced spontaneously at the accident site or reduced in the emergency department [4]. Neglected knee dislocations are not commonly seen, as the severity of the injury always leads to an early diagnosis [7].

Vincente-Guillen [8] reported a 15-year-old posterior knee dislocation treated with open reduction and external fixation, and finally arthrodesis of the joint. Sisto and Warren [9] described a case of chronic knee dislocation, treated 24 weeks after the acute injury with a reduction, and fixation with Steinmann crossed wires and pins.

While recent literature suggests a staged and early repair of the ligaments produces better results than late repair, there is no much information available on management of neglected dislocation [10]. The treatment options include open reduction and fixation with Steinman pins, external fixators, arthrodesis, and total knee arthroplasty [4], [11]. The purpose is to achieve a painless, mobile and stable knee joint without much functional disabilities. Staged reduction followed by reconstruction of ligaments has been described previously for an unreduced posterior dislocation of knee of up to 6 months of duration. For older dislocations, arthroplasty and arthrodesis are the two conventional modes of treatment [10], [11].

Arthrodesis is instead necessary in the presence of severe instability, paralysis, neuropathy, infections, and damage to the extensor mechanism. Although arthrodesis can cause persistent knee pain, lower back pain and limitations in resuming working activities [7]. There are a few reports on arthroplasty for chronic posterior knee dislocation. Constrained or semiconstrained prosthesis and quadriceps plasty are common recommendations [12].

for this patient we considered arthrodesis to be the most appropriate management because of the damaged cartilage of the articular surfaces and the presence of exuberant ectopic bone which hampered reduction.

## 4 Conclusion

Chronic knee dislocation is rare, as the severity of the injury always leads to an early diagnosis. However, dislocation of the knee, can be undiagnosed because of a diagnostic mistake at the time of the trauma, particularly in the absence of neurovascular injuries. Only a high clinical suspicion can avoid to neglect the more severe joint injury.

## Sources of funding

No sources of funding to declare.

## Ethical approval

This research did not require ethical approval due to the institute not requiring it for this type of study.

## Consent

Written informed consent was obtained from the patient for publication of this case report and accompanying image.

## Guarantor

Dr Hatim Garnaoui

## Provenance and peer review

Not commissioned, externally peer-reviewed.

## CRediT authorship contribution statement

Hatim Garnaoui: Corresponding author writing the paper.

Mohamed ali Trafeh: study concept.

Charafeddine El kassimi: supervised the writing of the manuscript.

Abderrahim Rafaoui: revision of paper.

Mohamed Rahmi: study concept.

Abdelhak Garch: correction of the paper.

## Declaration of competing interest

The authors declare having no conflicts of interest for this article.
